# The Correlation Between Internet Addiction and Interpersonal Relationship Among Teenagers and College Students Based on Pearson's Correlation Coefficient: A Systematic Review and Meta-Analysis

**DOI:** 10.3389/fpsyt.2022.818494

**Published:** 2022-03-10

**Authors:** Qing-hong Hao, Wei Peng, Jun Wang, Yang Tu, Hui Li, Tian-min Zhu

**Affiliations:** ^1^School of Rehabilitation and Health Preservation, Chengdu University of Traditional Chinese Medicine, Chengdu, China; ^2^School of Acupuncture and Tuina, Chengdu University of Traditional Chinese Medicine, Chengdu, China; ^3^School of Preclinical Medicine, Chengdu University, Chengdu, China

**Keywords:** internet addiction, interpersonal relationship, correlation, Pearson's correlation coefficient, meta-analysis

## Abstract

**Background:**

Internet addiction (IA) has become a serious social issue, inducing troubles in interpersonal relationships, which may negatively impact the healthy development of teenagers and college students. Thus, the current research aimed to synthesize the available evidence to clarify the correlation between IA and troubles in interpersonal relationships.

**Method:**

We searched eight electronic databases from inception to December 2020. Study quality was assessed by the Newcastle-Ottawa Scale (NOS), and Agency for Healthcare Research and Quality (AHRQ). We analyzed the data by extracting the Pearson correlation coefficients of each study and converted it into Fisher's Z. Pooled *r* was conducted by Fisher's Z and standard error (S_E_). STATA (Version 15.0) software was used for data synthesis.

**Results:**

A total of 10,173 studies were initially identified, and 26 studies (*n* = 14,638 participants) were retrieved for further analysis. The results indicated that there was a significant positive correlation between IA and interpersonal relationship troubles [0.36 (95% CI 0.35–0.38)]. In addition, there was a positive correlation between IA and different dimensions of interpersonal relationship reflected by troubles with interpersonal conversation, making friends, dealing with people, and heterosexual communication, with the result of [0.26 (95% CI 0.18–0.33)], [0.29 (95% CI 0.20–0.37)], [0.27 (95% CI 0.19–0.34)], [0.22 (0.15–0.30)], respectively. The Egger test suggested that there was no publication bias (*P* > 0.05).

**Conclusion:**

IA is positively correlated with troubles in interpersonal relationships. This research will provide new ideas and direction for further intervention, clinical therapy, and policy-making regarding IA to some extent.

**Systematic Review Registration:**

https://www.crd.york.ac.uk/prospero/, identifier: CRD42020177294.

## Introduction

Internet addiction (IA), described as a behavioral addiction, manifested as repeated and unrestrained use of the internet, induces a series of cognitive and social impairments (1–3). With the advancement of the internet, IA is widely prevelant among teenagers in many countries around the world, reaching a maximum of 57.5% (4–6). Teenagers and college students are the main groups who use the internet, and among all internet devices, mobile phones are the main tool for them to surf the internet (7). In modern society, the internet is certainly important, but a series of public health problems caused by it cannot be ignored. At present, many studies have shown that IA is related to the troubles in interpersonal relationships among teenagers and college students (8, 9).

Interpersonal relationships are a phenomenon that exists only in human society, and there is no unified definition at present (10). Some scholars believe that definitions of interpersonal relationships emphasize a mutual process, that is, a bond among people that arises in the process of mutual contact due to common needs (11, 12). The Interpersonal Comprehensive Diagnostic Scale (ICDS) compiled by Zheng (13) is divided into four dimensions (trouble with interpersonal conversation, making friends, dealing with people, heterosexual communication), which can better reflect the interpersonal relationship troubles of teenagers and college students. Adolescence is a critical period for physical and mental development, during which the development of positive interpersonal relationships is the foundation for entry into society (8). On the contrary, if effective interpersonal relationships are not developed, various psychological issues will arise, such as depression, anxiety, aggression, and maladjustment (14–16). What is more, many studies have found that IA is an important factor influencing interpersonal relationships (9, 17, 18). This is probably due to the convenience, anonymity, and virtuality of the internet being more likely to arouse their attention (19). Therefore, it is necessary to investigate the correlation between IA and troubles in interpersonal relationships among teenagers and college students.

There have been many related studies discussing the relationship between IA and interpersonal relationship troubles. The study by Lo et al. (20) about 174 online gamers among Taiwanese college students found that with increased time spent in game, issues with interpersonal relationships appeared, and social anxiety increased. The research by Wang et al. (21) pointed out that the degree of mobile phone addiction is slight, but their outlook for interpersonal troubles was not optimistic. At the same time, this study also showed that there was an obvious positive correlation between mobile phone addiction and the interpersonal troubles of college students. However, the study by Li (22) indicated that IA had a weak effect on interpersonal relationships. A study by Dai (23) showed that the interpersonal relationship and IA of teenagers were not significantly related. Otherwise, in terms of different dimensions of interpersonal relationships, the study by Zhang (24) believed that heterosexual communication and interpersonal conversation were significantly related to IA. However, the results of different studies were not consistent (25, 26). Therefore, for these inconsistent results, we chose a meta-analysis to summarize the current studies with the aim of obtaining a consistent finding.

## Materials and Methods

This systematic review was conducted in accordance with Preferred Reporting Items for Systematic Reviews and Meta-Analyses (PRISMA) (27). The systematic review protocol has been registered in PROSPERO with registration number is CRD42020177294.

### Literature Search

PubMed, Embase, Medline, Web of Science, China National Knowledge Infrastructure (CNKI), China Biology Medicine (CBM), Wan Fang data, and the Chinese Science and Technology Periodical Database (VIP) were be searched for relevant studies in Chinese and English before December 2020. In addition to the electronic databases, we also searched conference papers, dissertations, and reference lists of relevant reviews, so as to ensure the integrity of the inclusion of the literature. The search strategy is shown in Supplementary Material 1.

All retrieved literature was managed using Endnote X9 and repeated records were filtered. Two authors (QH and JW) independently screened the articles according to the title and abstract based on the pre-set inclusion criteria. They then downloaded the full text of all possibly relevant studies, and further reviewed the full text independently. They cross-examined the included studies, and the differences were resolved through discussion or consensus with third-party reviewers (WP).

### Selection of Articles

#### Types of Studies

All observational studies including cohort, case-control, and cross-sectional studies were included if a correlation between IA and interpersonal relationship troubles were reported as an outcome before December 2020. And all studies provided effective Pearson correlation coefficients. Other types of studies including reviews, case reports, meeting abstracts, and commentaries were excluded.

#### Types of Participants

The study participants were teenagers and college students who were diagnosed with IA. No limitation was exerted upon gender.

#### Types of Intervention and Comparison

Because this study was a correlation meta-analysis, it did not involve grouping. So, there is no strict setting of the intervention and comparison group.

#### Types of Outcomes

The main outcomes were the scales related to IA and interpersonal relationships. Pearson correlation coefficients reported by each study was used to further analyze the correlation between IA and troubles in interpersonal relationship.

The secondary outcomes were the correlation analysis between different dimensions of interpersonal relationships and IA.

### Data Extractions

Two authors (QH and JW) independently extracted the data with a pre-defined data extraction form, including general characteristics (first author, year of publication, language, and research country/region), characteristics of participants (sample size, average age, sex, study population), other characteristics (IA diagnostic criteria, measurement tools used, interpersonal categories) and outcomes (scale scores, correlation coefficient between them and *P*-value, main results). Of course, it also included more information related to this study.

### Quality Assessment

In this study, the Newcastle-Ottawa Quality Assessment Form (NOS) (28) was utilized to access the quality of the case-control studies, which included three quality parameters (selection, comparability, and results). The Agency for Healthcare Research and Quality (AHRQ) (29) was utilized to access the cross-sectional studies, including 11 items. The score of 0–3 was classified as low quality, 4–7 was classified as medium quality, and 8–11 was classified as high quality. All stages of the quality assessment process were independently performed by two authors (QH and JW). The differences were resolved through discussion or consensus with third-party reviewers (YT).

### Data Analysis

We extracted the Pearson correlation coefficients (*r*) of each study and conducted a meta-analysis about the correlation between IA and troubles in interpersonal relationships among teenagers and college students. After converting the Pearson correlation coefficients (*r*) into Fisher's Z and SE, the final effect size was calculated as the pooled r and 95% confidence interval (95% CI). The same methods were used to analyze the correlation between IA and different dimensions of interpersonal relationship. The heterogeneity of the *r*-values between studies was determined by calculating the Q statistic, which derived from the chi-square test, and the inconsistency index (*I*^2^) (30, 31). If *p* < 0.05 or *I*^2^ > 50%, the heterogeneity was indicated (32). If significant heterogeneity was found, sensitivity analyses were performed on all studies to further investigate the heterogeneity of the studies. In addition, publication bias was conducted by funnel plot. Besides, Egger's test was to be carried out. All procedures were executed by STATA (Version 15.0) software.

## Results

### Literature Search

**Figure 1:**
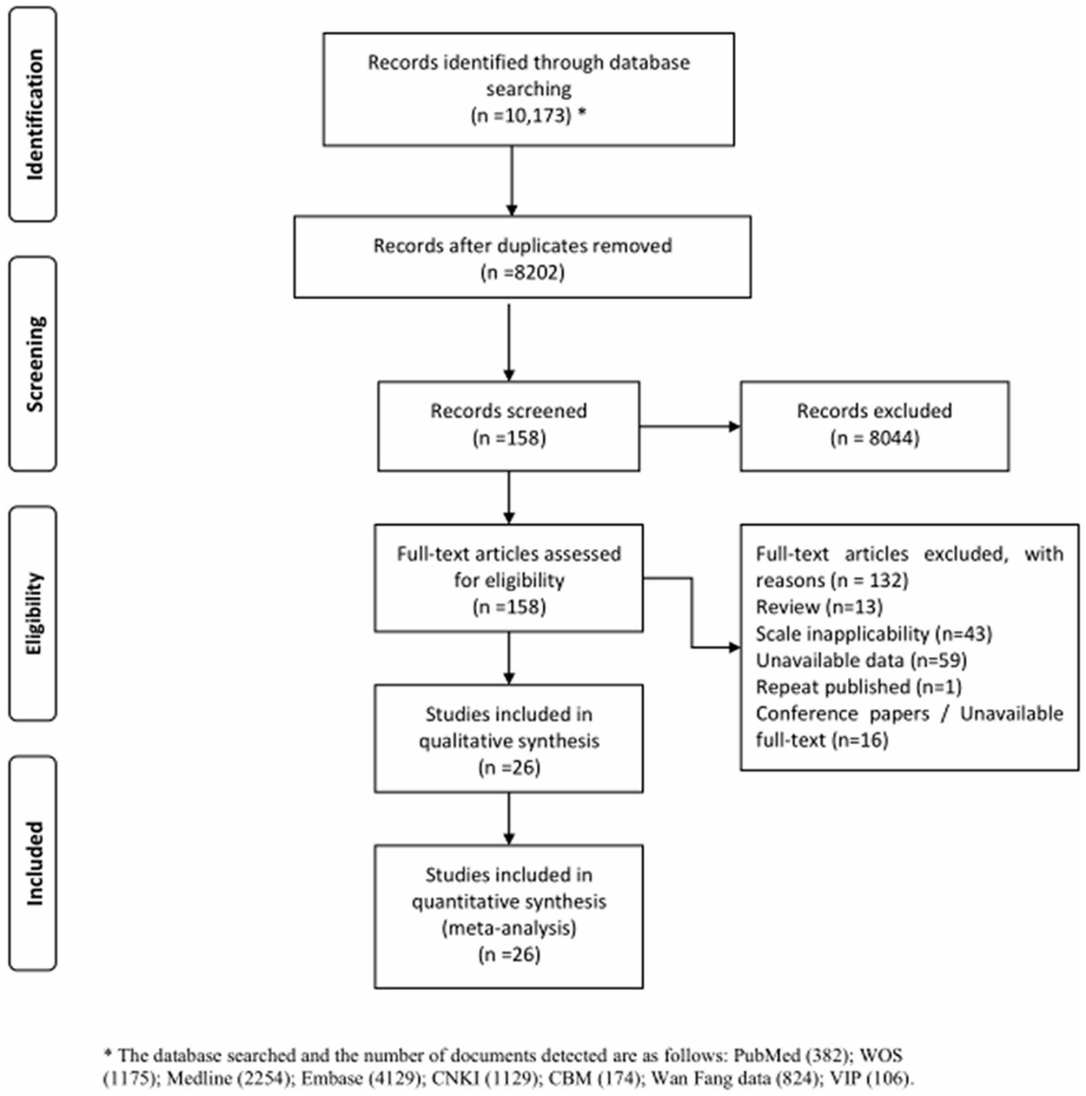
PRISMA flow diagram.

Through searching the database using our search strategy, 10,173 studies were initially identified, and 8,202 studies remain after deduplication with Endnote X9. After screening the titles and abstracts, a total of 158 studies required full-text evaluation. Finally, 26 studies were included in this systematic review and meta-analysis. The detailed screening process was shown in a PRISMA flow diagram (see Figure 1).

### Study Characteristics

A total of 26 studies were included in this review, with a total of 14,638 participants. The studies included in this study were observational studies, including 19 cross-sectional studies (8, 21, 24, 33–48) and 7 case-control studies (49–55). Regarding the tools used to assess IA, five studies (33, 34, 37, 39, 49) used Revised Chen Internet Addiction Scale (CIAS-R), four studies (35, 36, 48, 55) used Young Internet Addiction Test (YIAT), two studies (24, 38) used Chen Internet Addiction Scale (CIAS), two studies (43, 45) used Mobile Phone Addiction Index (MPAI), 1 study (50) used Internet Addiction Impairment Indexes (IAII), nine studies (21, 41, 42, 45, 46, 48, 51, 52, 54) used Mobile Phone Addiction Tendency Scale (MPATS), one study (40) used Mobile Phone Dependence Scale (MPDS), one study (53) used Adolescent Pathological Internet Use Scale (APIUS), and one study (8) used the Korean version of Internet Addiction self-test scale. Regarding the evaluation of interpersonal relationships, except for the study by Seo et al. (8), which used the Korean version “Inventory of Interpersonal Problems,” almost all studies used the Interpersonal Comprehensive Diagnostic Scale (ICDS). The basic characteristics of the included studies were summarized in Table 1.

**Table 1:** The basic characteristics of the included studies.

**References**	**Region**	**Sample size (Male/Female)**	**Age range/Population**	**Study design**	**Assessment scales**		**r**	***P***	**Study quality scores**
					**Internet addiction**	**Interpersonal relationship**			
Liu (33)	Wuhan	341 (169/172)	College students	Cross-sectional	CIAS-R	ICDS	0.397	<0.01	5
Wang et al. (34)	Anhui	1,232 (806/426)	College students	Cross-sectional	CIAS-R	ICDS	0.381	<0.01	5
Zhang (a) et al. (35)	Anhui	177 (88/89)	College students	Cross-sectional	YIAT	ICDS	0.238	<0.01	6
Zhang (b) et al. (24)	Jiangsu	389 (226/163)	18–24	Cross-sectional	CIAS	ICDS	0.356	<0.001	6
Chen et al. (36)	Shandong	368 (191/177)	College students	Cross-sectional	YIAT	ICDS	0.32	<0.01	6
Zheng et al. (49)	Guangdong	657 (289/368)	17–23/college students	Case-control	CIAS-R	ICDS	0.42	<0.01	6
Zhang et al. (37)	Beijing	176 (92/84)	20–29/college students	Cross-sectional	CIAS-R	ICDS	0.375	<0.01	5
Yuan (50)	Hunan	832 (372/460)	14–19/middle school students	Case-control	IAII	ICDS	0.336	<0.01	7
Wu et al. (38)	Dalian	201 (94/107)	College students	Cross-sectional	CIAS	ICDS	0.227	<0.05	5
Liu et al. (39)	Hebei	490	College students	Cross-sectional	CIAS-R	ICDS	0.470	<0.01	6
Yang (51)	Shandong	723 (285/438)	College students	Case-control	MPATS	ICDS	0.29	<0.01	7
Liao et al. (52)	Jiangxi	472 (218/254)	18–25/college students	Case-control	MPATS	ICDS	0.349	<0.01	7
Wang et al. (21)	Guangxi	348	College students	Cross-sectional	MPATS	ICDS	0.382	<0.01	5
Ye (40)	Anhui	871 (538/333)	College students	Cross-sectional	MPDS	ICDS	0.333	<0.001	7
Tan (53)	Hunan	524 (193/331)	College students	Case-control	APIUS	ICDS	0.403	<0.01	7
Liu et al. (54)	Sichuan	200 (73/127)	College students	Case-control	MPATS	ICDS	0.345	<0.001	7
Lai (41)	Jiangxi	560 (265/295)	17–24/college students	Cross-sectional	MPATS	ICDS	0.349	<0.01	6
Tang et al. (42)	Guangxi	780 (376/404)	College students	Cross-sectional	MPATS	ICDS	0.32	<0.01	7
Aruna (44)	Heilongjiang	513 (339/174)	College students	Cross-sectional	MPAI	ICDS	0.414	<0.001	7
Ma et al. (46)	Henan	798 (204/594)	College students	Cross-sectional	MPATS	ICDS	0.329	<0.001	7
Su et al. (48)	Shanxi	943	18–22/college students	Cross-sectional	MPATS	ICDS	0.394	<0.01	8
Kong et al. (47)	Guizhou	479 (168/311)	College students	Cross-sectional	YIAT	ICDS	0.31	<0.01	6
He (45)	Zhejiang	1,075 (480/595)	Middle school students	Cross-sectional	MPATS	ICDS	0.37	<0.01	6
Seo et al. (8)	Seoul, Korea	676 (378/297)	12–17/middle school students	Cross-sectional	IAT (Korean version)	The Inventory of Interpersonal Problems (Korean version)	0.425	<0.001	6
Cen et al. (55)	Guizhou	403 (198/205)	21.05 ±1.04/college students	Case-control	YIAT	ICDS	0.776	<0.01	7
Wang (43)	Guangdong	410 (156/254)	13.46 ± 1.22/middle school students	Cross-sectional	MPAI	ICDS	−0.323	<0.01	7

*CIAS-R, revised chen internet addiction scale; YIAT, the young internet addiction test; CIAS, chen internet addiction scale; IAII, internet addiction impairment indexes; MPATS, mobile phone addiction tendency scale; APIUS, adolescent pathological internet use scale; MPAI, mobile phone addiction index; MPDS, mobile phone dependence scale; APIUS, adolescent pathological internet use scale; ICDS, interpersonal comprehensive diagnostic scale*.

### Methodological Quality

In this review, the AHRQ scale was used to evaluate cross-sectional studies, and the NOS scale was used to evaluate case-control studies. Through the evaluation, we found that the overall quality of included studies was good, and there were no low-quality studies. The detailed information about methodological quality assessment was presented in Tables 2, 3.

**Table 2:** Risk assessment results of bias included in cross-sectional studies (score).

**References**	**(1)**	**(2)**	**(3)**	**(4)**	**(5)**	**(6)**	**(7)**	**(8)**	**(9)**	**(10)**	**(11)**	**Scores**
Liu (33)	1	1	0	1	0	1	0	0	0	1	0	5
Wang et al. (34)	1	1	0	1	0	1	0	0	0	1	0	5
Zhang (a) et al. (35)	1	1	0	1	0	1	0	1	0	1	0	6
Zhang (b) et al. (24)	1	1	0	1	0	1	0	1	0	1	0	6
Chen et al. (36)	1	1	0	1	0	1	0	1	0	1	0	6
Zhang et al. (37)	1	1	0	1	0	1	0	0	0	1	0	5
Wu et al. (38)	1	1	0	1	0	1	0	0	0	1	0	5
Liu et al. (39)	1	1	1	1	0	1	0	0	0	1	0	6
Wang et al. (21)	1	1	0	1	0	1	0	0	0	1	0	5
Ye (40)	1	1	0	1	0	1	1	1	0	1	0	7
Lai (41)	1	1	0	1	0	1	0	1	0	1	0	6
Tang et al. (42)	1	1	1	1	0	1	0	1	0	1	0	7
Aruna (44)	1	1	0	1	0	1	1	1	0	1	0	7
Ma et al. (46)	1	1	1	1	0	1	0	1	0	1	0	7
Su et al. (48)	1	1	1	1	0	1	1	1	0	1	0	8
Kong et al. (47)	1	1	0	1	0	1	0	1	0	1	0	6
He (45)	1	1	0	1	0	1	0	1	0	1	0	6
Seo et al. (8)	1	1	0	1	0	1	0	1	0	1	0	6
Wang (43)	1	0	1	1	0	1	1	1	0	1	0	7

*(1) Define the source of information (survey, record review)? (2) List inclusion and exclusion criteria for exposed and unexposed subjects (cases and controls) or refer to previous publications? (3) Indicate time period used for identifying patients? (4) Indicate whether or not subjects were consecutive if not population-based? (5) Indicate if evaluators of subjective components of the study were masked to other aspects of the status of the participants? (6) Describe any assessments undertaken for quality assurance purposes (e.g., tests/retest of primary outcome measurements)? (7) Explain any patient exclusions from analysis? (8) Describe how confounding was assessed and/or controlled? (9) If applicable, explain how missing data were handled in the analysis? (10) Summarize patient response rates and completeness of data collection? (11) Clarify what follow-up, if any, was expected and the percentage of patients for which incomplete data or follow-up was obtained?*

**Table 3:** Risk assessment results of bias included in case-control studies (score).

	**Selection**				**Comparability**	**Exposure**			**Scores**
	**(1)**	**(2)**	**(3)**	**(4)**	**(5)**	**(6)**	**(7)**	**(8)**	
Zheng et al. (49)	1	1	1	1	1	0	1	0	6
Yuan (50)	1	1	1	1	2	0	1	0	7
Yang (51)	1	1	1	1	2	0	1	0	7
Liao et al. (52)	1	1	1	1	2	0	1	0	7
Tan (53)	1	1	1	1	2	0	1	0	7
Liu et al. (54)	1	1	1	1	2	0	1	0	7
Cen et al. (55)	1	1	1	1	2	0	1	0	7

*(1) Is the case definition adequate? (2) Representativeness of the case. (3) Selection of controls. (4) Definition of controls. (5) Comparability of cases and controls on the basis of design or analysis. (6) Ascertainment of exposure. (7) Same method of ascertainment for cases and controls. (8) Non-response rate*.

### Meta-Analysis Results

By extracting the correlation coefficient between IA and interpersonal relationships in different studies, and after appropriate conversion, we conducted a meta-analysis of included studies. Knowing from the forest plot, the pooled effect size (*z*) was 0.37 (95% CI 0.30–0.44). After conversion, the pooled *r* was 0.36 (95% CI 0.30–0.41), and there was significant heterogeneity among studies (*I*^2^ = 94.1%, *P* < 0.001).

### Sensitivity Analysis

**Figure 2:**
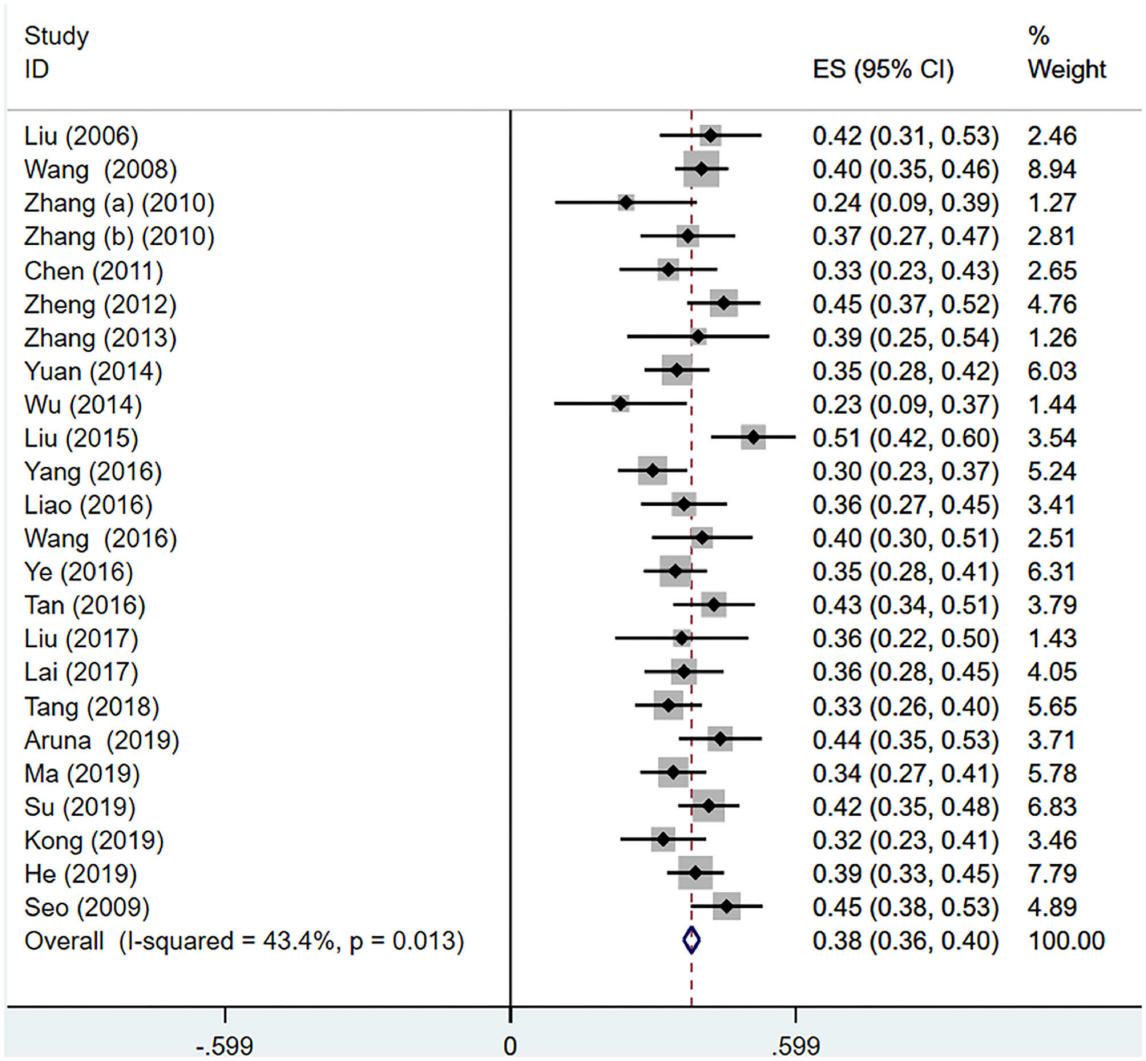
The correlation between IA and interpersonal relationships.

**Figure 3:**
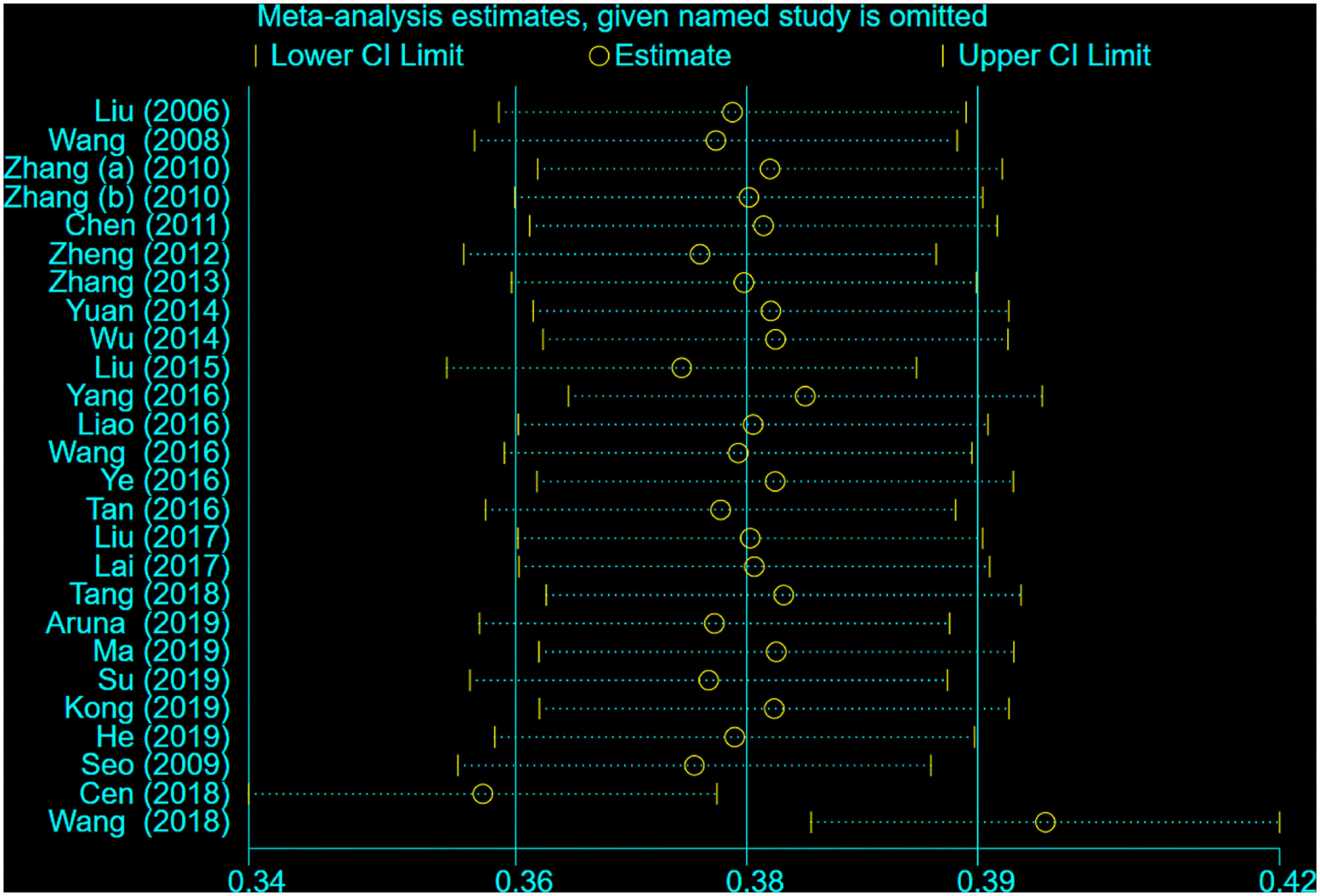
Sensitivity analysis of each study.

The sensitivity analysis was carried out to discovered the source of heterogeneity. We found that the studies of Wang (43) and Cen (55) were the source of high heterogeneity. Then, we recalculated the pooled r after removing these two studies, the pooled *r* was 0.36 (95% CI 0.35–0.38), and there was no significant heterogeneity (*I*^2^ = 43.4%, *P* < 0.05; see Figure 2). The result of sensitivity analysis is shown in Figure 3.

### Publication Bias

**Figure 4:**
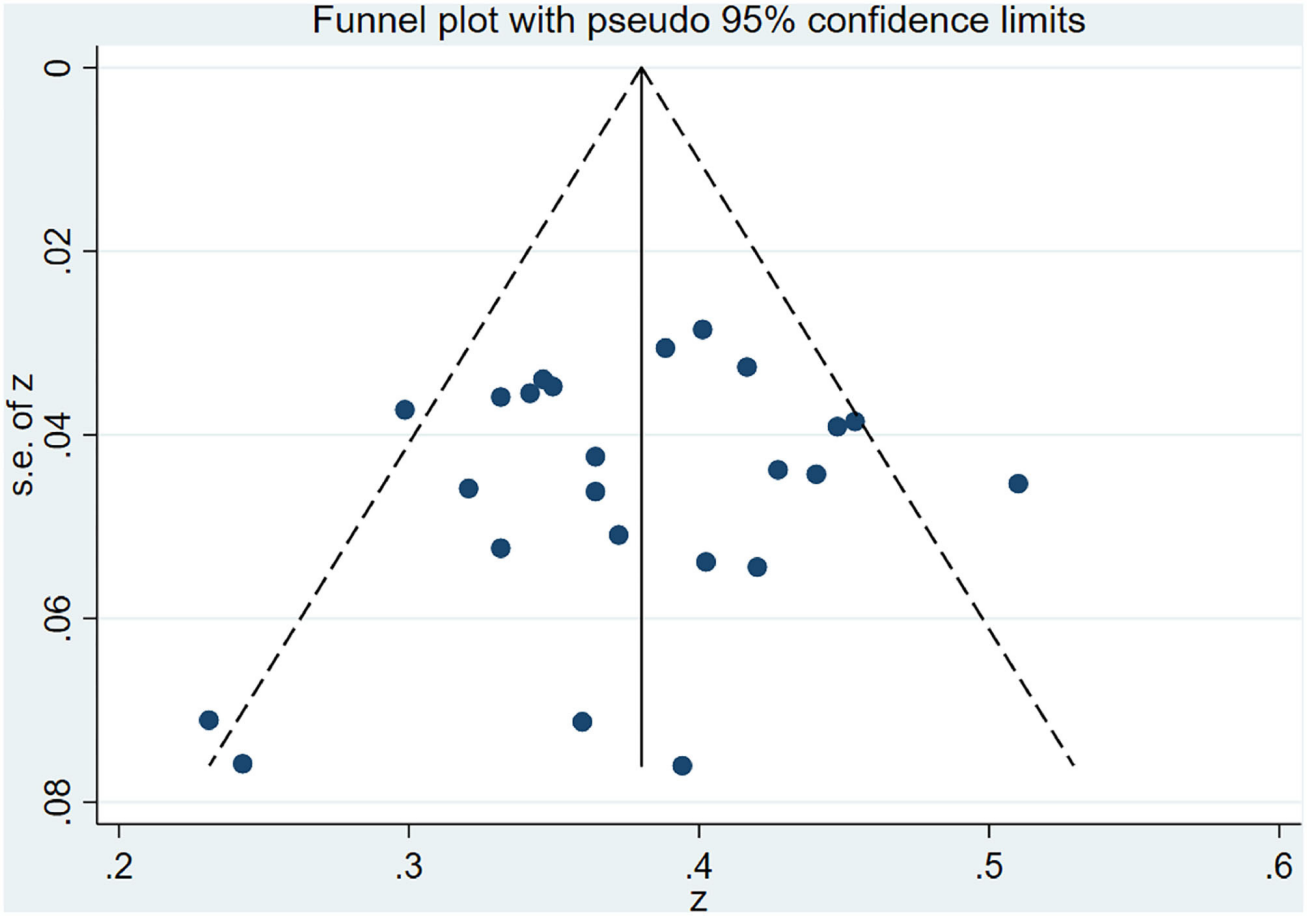
The funnel plot of publication bias.

After removing two high heterogeneity studies, the funnel plot was basically symmetrical (see Figure 4). The Egger's test suggested that there was no publication bias (*P* > 0.05).

### Meta-Analysis Between IA and Different Dimensions of Interpersonal Relationship

We analyzed the statistically significant data that provided different factors' coefficients of ICDS in the included studies. Through analysis, we found that there was some heterogeneity among studies. However, through sensitivity analysis and after removing some studies, we found that the overall effect size did not change substantially (*I*^2^ > 50%), so we chose the random-effects model. In addition, the results should be treated with caution due to their high heterogeneity.

#### IA and Trouble With Interpersonal Conversation

**Figure 5:**
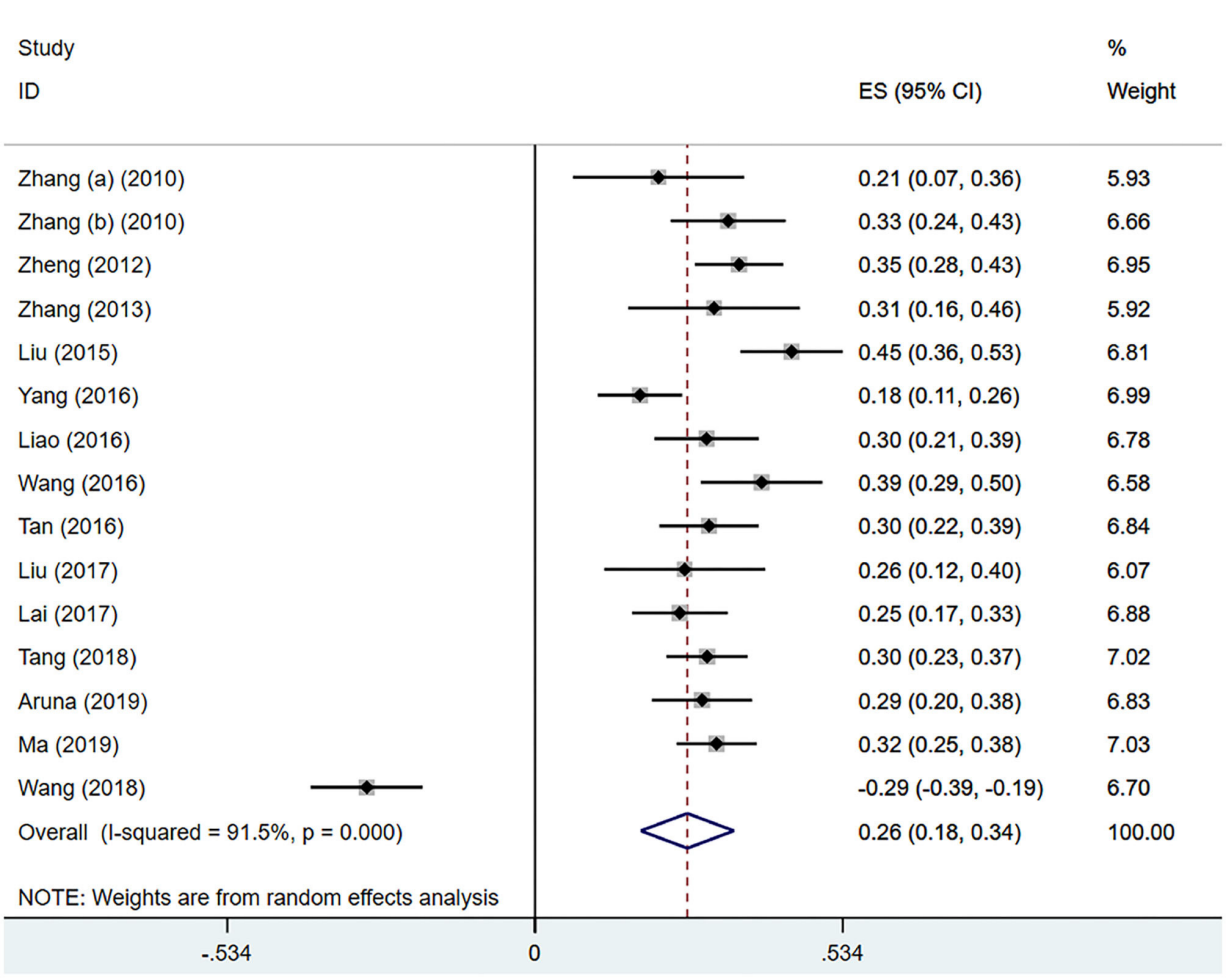
IA and trouble with interpersonal conversation.

Fifteen (21, 24, 35, 37, 39, 41–44, 46, 49, 51–54) studies mentioned the meaningful correlation coefficients of interpersonal conversation trouble factors. Through analysis, there were a large heterogeneity (*I*^2^ = 91.5%, *P* < 0.001), and the pooled effect size (*z*) was 0.26 (95% CI 0.18–0.34; Figure 5). After conversion, the pooled *r* was 0.26 (95% CI 0.18–0.33; Table 4).

**Table 4:** The pooled effect size (*r*) after conversion.

**Factors**	**Pooled r**	**95%CI**	***I* ^2^**	***P***
Trouble with interpersonal conversation	0.26	(0.18, 0.33)	91.5%	<0.001
Trouble with making friends	0.29	(0.20, 0.37)	94.7%	<0.001
Trouble dealing with people	0.27	(0.19, 0.34)	93.1%	<0.001
Trouble with heterosexual communication	0.22	(0.15, 0.30)	92.6%	<0.001

#### IA and Troubled With Making Friends

**Figure 6:**
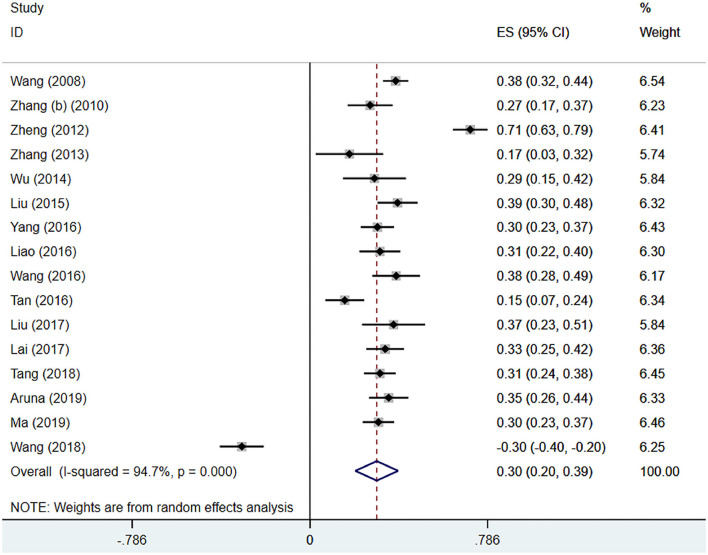
IA and trouble with making friends.

Sixteen (21, 24, 34, 37–39, 41–44, 46, 49, 51–54) studies mentioned the meaningful correlation coefficients of trouble with making friends. Through analysis, there was a large heterogeneity (*I*^2^ = 94.7%, *P* < 0.001), the pooled effect size (*z*) was 0.30 (95% CI 0.20–0.39; Figure 6). After conversion, the pooled *r* was 0.29 (95% CI 0.20–0.37; Table 4).

#### IA and Trouble Dealing With People

**Figure 7:**
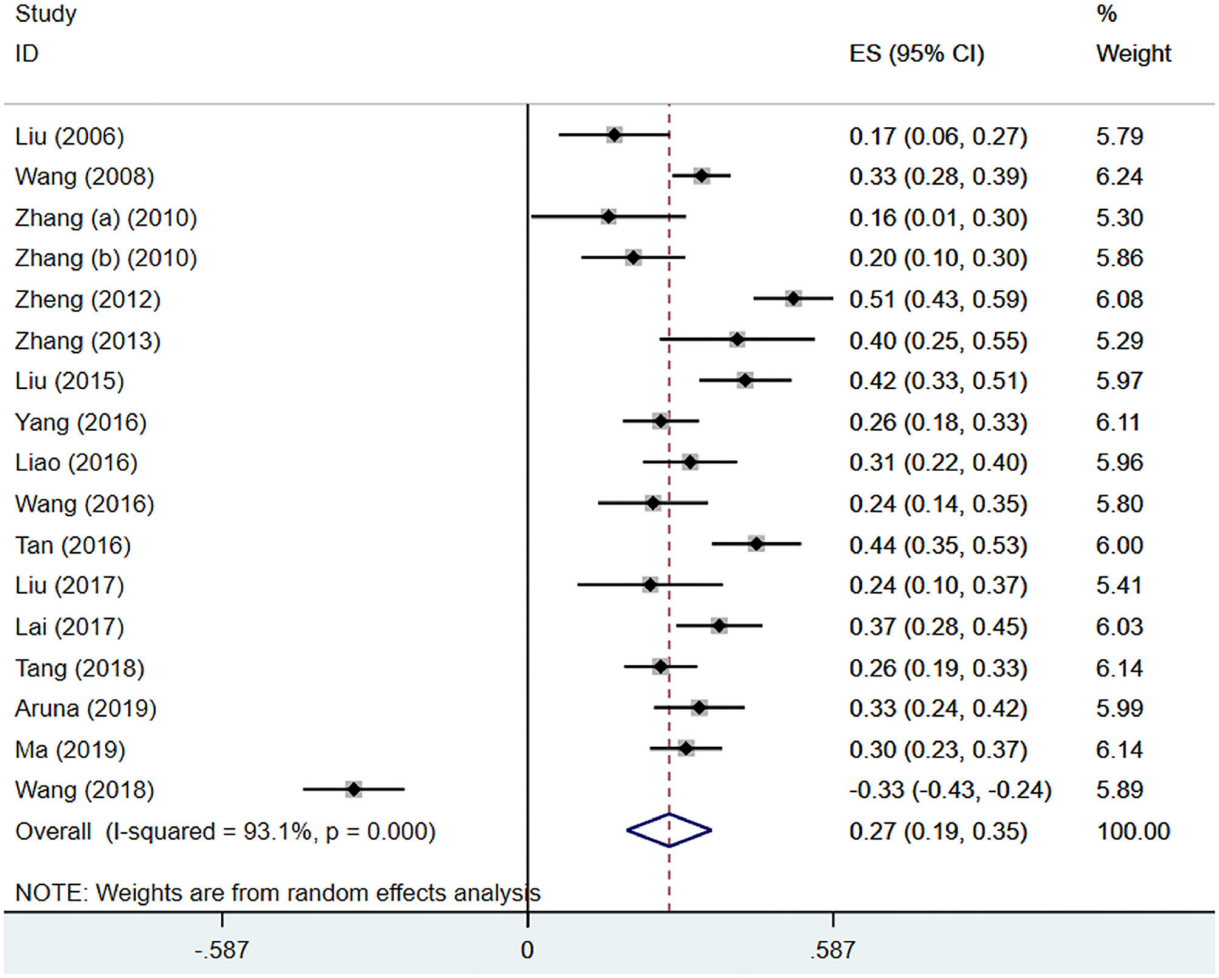
IA and trouble dealing with people.

Seventeen (21, 24, 33–35, 37, 39, 41–44, 46, 49, 51–54) studies mentioned the meaningful correlation coefficients of trouble dealing with people. Through analysis, there was significant heterogeneity (*I*^2^ = 93.1%, *P* < 0.001), the pooled effect size (*z*) was 0.27 (95% CI 0.19–0.35; Figure 7). After conversion, the pooled *r* was 0.27 (95% CI 0.19–0.34; Table 4).

#### IA and Trouble With Heterosexual Communication

**Figure 8:**
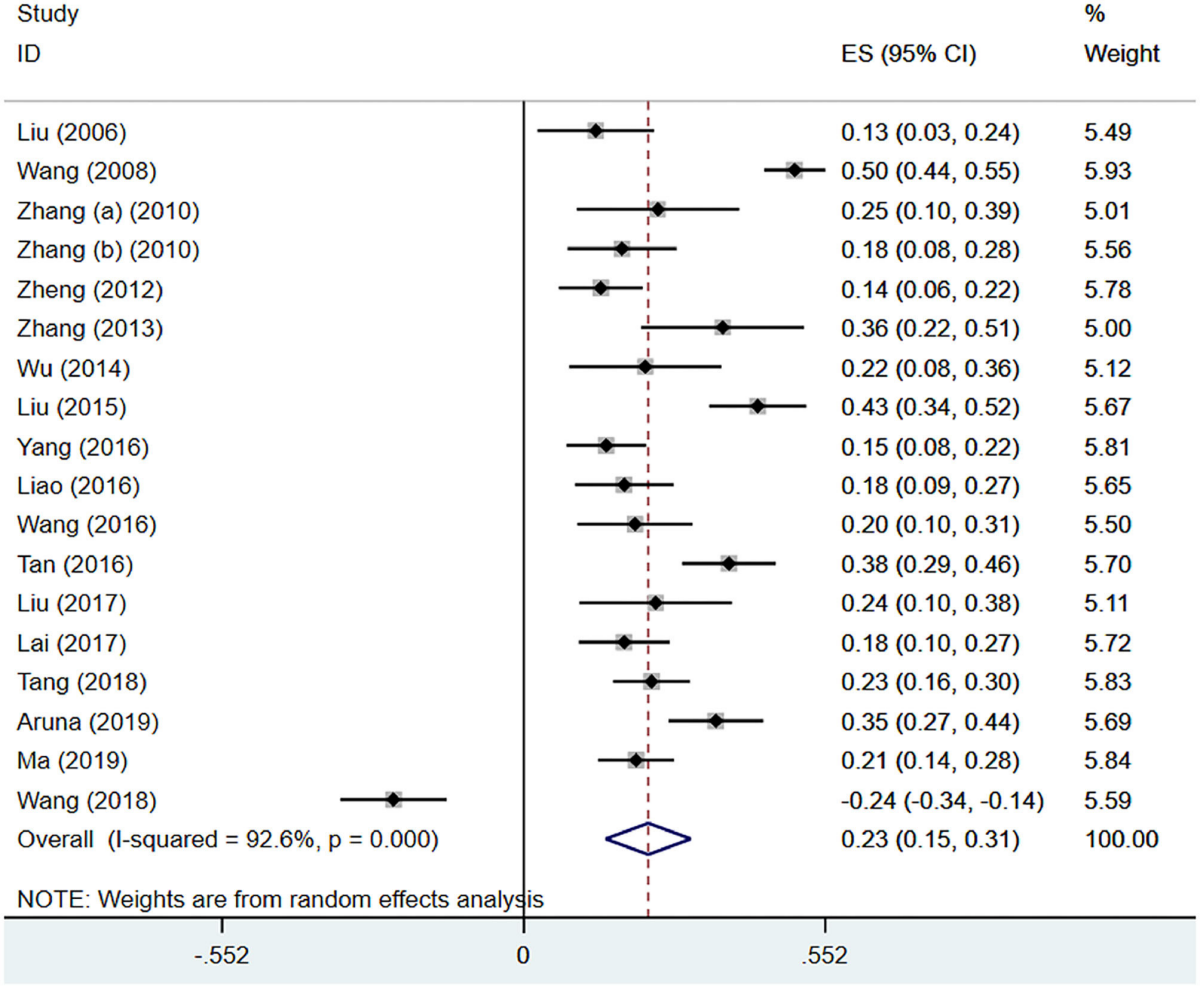
IA and trouble with heterosexual communication.

Eighteen (21, 24, 33–35, 37–39, 41–44, 46, 49, 51–54) studies mentioned the meaningful correlation coefficients of trouble with heterosexual communication. Through analysis, there was a large heterogeneity (*I*^2^ = 92.6%, *P* < 0.001), the pooled effect size (*z*) was 0.23 (95% CI 0.15–0.31; Figure 8). After conversion, the pooled *r* was 0.22 (95% CI 0.15–0.30; Table 4).

## Discussion

In this study, we extracted the Pearson correlation coefficients of each study to explore the correlation between IA and troubles in interpersonal relationships, in order to get a consistent result. To the best of our knowledge, this is the first meta-analysis to investigate the association between IA and troubles in interpersonal relationships with consideration of heterogeneity. The findings revealed that there is a positive correlation between IA and interpersonal relationship troubles. Moreover, the dimension of making friends showed a stronger correlation with IA than the other three dimensions.

Through meta-analysis, we found that IA has an obvious positive correlation with interpersonal relationship troubles in teenagers and college students. This is consistent with most current research results (56, 57). The study by Qiao et al. (58) pointed out that the current status of interpersonal relationship was an important factor influencing the degree of IA. Contemporary teenagers and college students relying on the internet for a long time, where they can exchange information through unsentimental symbols, would aggravate their real interpersonal troubles (59). The reason for this might be that over-reliance on the internet takes up most of their time, leading to reduced social participation and distant interpersonal relationships. Moreover, among the factors influencing IA and interpersonal relationships, many of them play a mediating role, such as loneliness, self-esteem, shyness, anxiety, and pressure. However, relevant studies are limited and the data do not support further analysis.

As mentioned in the results, we removed the two studies by Cen (55) and Wang (43) due to high heterogeneity. The study by Cen (55) divided IA into non-internet addicted group, slight addiction group, and severe addiction group, while other studies did not. The classification of IA affected the scores of the IA scale, which may lead to some bias in the results of Pearson's correlation analysis. The study by Wang (43) found a significant negative correlation between IA and interpersonal relationships, which was too different from other study results. We guess this might be because it did not clearly describe randomization and inclusion criteria as other studies did. Therefore, according to the above reasons, we chose to remove these two studies in order to get more accurate and reliable conclusions.

What is more, the correlation between different dimensions of interpersonal relationships and IA was different. Study results showed that there was a significant positive correlation between IA and different dimensions of interpersonal relationships. Compared with the other three dimensions, the dimension of making friends showed a stronger correlation with IA. This may be because teenagers and college students with interpersonal troubles are more inclined to make friends through the internet (60), while in reality, there would be much less interaction with others.

In general, all dimensions were correlated with IA. First of all, for teenagers and college students with interpersonal troubles in conversational behavior, some people who are not good at talking have less communication with others in reality, and they tend to use the internet to dispel inner anxiety when encountering setbacks and difficulties. Over time, social barriers will appear, they may become more reliant on the internet and even develop into IA (39). Secondly, teenagers and college students with a poor ability to deal with others are not good at expressing themselves and do not know how to deal with complex relationships in life, which leads to tension in their real interpersonal relationships (54). On the internet, there is no face-to-face communication, they can express their opinions more casually, be themselves truly, and even gain a sense of identity through the internet. But this kind of communication on the internet can not improve but hinder and suppress their real ability to deal with others. Finally, in terms of communicating with the opposite sex, some people who are nervous about communicating with them in real life do not find it easy to get attention from the opposite sex (33, 61). On the internet, they can be more relaxed and natural without too much worry. Therefore, they prefer to seek comfort in the virtual online world.

In summary, IA has a positive correlation with interpersonal troubles, especially in the dimension of making friends. Adolescence is an important growth stage for teenagers, so families and schools should pay attention to their mental health education, and strive to guide them to better deal with the relationship between interpersonal and internet, to help them improve their interpersonal communication skills, and better adapt to the society. Moreover, the problem of IA has aroused widespread concern in society, and it has been introduced as a risk factor influencing the physical and mental health of teenagers and college students (62). Therefore, the effect of IA on interpersonal disturbance is not negligible. Each family and school should pay attention, and prevent them from indulging in the internet and forming serious psychological crises of trust and personality disorder (63). Parents should give more care and support to their children, and schools also need to carry out more health education lectures to help teenagers and college students establish healthy concepts of interpersonal relationships.

There are several limitations to this study. Firstly, the IA scales used in each study were not identical, which may have a certain impact on the results. Secondly, the researches included in this study are almost Chinese studies, which is not quite clear about the IA and interpersonal relationships of teenagers and college students in other countries. Finally, there were many intermediary factors that affect IA and interpersonal relationships, but relevant data cannot be analyzed and summarized yet. In the future, more relevant studies may be needed to confirm the role of these intermediary factors.

## Conclusion

This review suggested that there is a positive correlation between IA and troubles in interpersonal relationships, with the dimension of making friends showing a stronger correlation than the other three aspects. Teenagers and college students are high-incidence groups of IA while developing appropriate interpersonal relationships is a cornerstone of growth for them. Thus, both families and schools should play an important role in guiding them to use the internet reasonably and establish satisfactory interpersonal relationships. The results of this study will provide new ideas and direction for further intervention, clinical therapy, and policy-making of IA to some extent.

## Data Availability Statement

The raw data supporting the conclusions of this article will be made available by the authors, without undue reservation.

## Author Contributions

QH: data curation, formal analysis, investigation, methodology, resources, software, and writing—original draft. WP: conceptualization, data curation, resources, software, and writing—review and editing. JW: data curation, formal analysis, methodology, and resources. YT: formal analysis, investigation, and writing—review and editing. HL and TZ: conceptualization, funding acquisition, project administration, and writing—review and editing. All authors contributed to the article and approved the submitted version.

## Funding

This research was supported by the Natural Science Foundation of China (81072852 and 81574047), the Key R&D Project of Sichuan Province (2019YFS0175), the Xinglin Scholar Research Promotion Project of Chengdu University of TCM (XSGG2019007), and the Training Funds of Academic and Technical Leader in Sichuan Province.

## Conflict of Interest

The authors declare that the research was conducted in the absence of any commercial or financial relationships that could be construed as a potential conflict of interest.

## Publisher's Note

All claims expressed in this article are solely those of the authors and do not necessarily represent those of their affiliated organizations, or those of the publisher, the editors and the reviewers. Any product that may be evaluated in this article, or claim that may be made by its manufacturer, is not guaranteed or endorsed by the publisher.
