# Supplementary material for: The Correlation Between Internet Addiction and Interpersonal Relationship Among Teenagers and College Students Based on Pearson's Correlation Coefficient: A Systematic Review and Meta-Analysis
Source: Front Psychiatry. 2022 Mar 10;13:818494. doi: 10.3389/fpsyt.2022.818494 (PMC8960053; doi:10.3389/fpsyt.2022.818494)
Supplement: Supplementary file 2 [file Data_Sheet_1.docx]

**The detailed Search terms:**

**PubMed**

("internet"[Mesh] OR "online"[tiab] OR "social media"[tiab] OR "smartphone"[tiab] OR "web"[tiab] OR " internet game "[tiab] OR " facebook "[tiab] AND "addiction"[tiab] OR "pathological"[tiab] OR "excessive"[tiab]) OR "disorder"[tiab]) OR "overuse"[tiab]) OR "problem"[tiab]) AND ("Interpersonal Relation"[Mesh] OR "Relation, Interpersonal"[tiab] OR "Relations, Interpersonal"[tiab] OR "Social Interaction"[tiab]) OR "Interactions, Social"[tiab]) OR "Social Interactions"[tiab]) OR "Partner Communication"[tiab] OR "Communication, Partner"[tiab]) OR "Communications, Partner"[tiab]) OR "Partner Communications"[tiab]) OR "peer relation"[tiab] OR "parent-child relation"[tiab]) OR "teacher-student relation"[tiab])
